# Network Inference With the Lasso

**DOI:** 10.1080/00273171.2024.2317928

**Published:** 2024-04-08

**Authors:** Lourens Waldorp, Jonas Haslbeck

**Affiliations:** Psychological Methods, University of Amsterdam, Amsterdam, the Netherlands

**Keywords:** *p*-values for lasso, multisplit method, bootstrap, desparsified lasso, debiased lasso

## Abstract

Calculating confidence intervals and *p*-values of edges in networks is useful to decide their presence or absence and it is a natural way to quantify uncertainty. Since lasso estimation is often used to obtain edges in a network, and the underlying distribution of lasso estimates is discontinuous and has probability one at zero when the estimate is zero, obtaining *p*-values and confidence intervals is problematic. It is also not always desirable to use the lasso to select the edges because there are assumptions required for correct identification of network edges that may not be warranted for the data at hand. Here, we review three methods that either use a modified lasso estimate (desparsified or debiased lasso) or a method that uses the lasso for selection and then determines *p*-values without the lasso. We compare these three methods with popular methods to estimate Gaussian Graphical Models in simulations and conclude that the desparsified lasso and its bootstrapped version appear to be the best choices for selection and quantifying uncertainty with confidence intervals and *p*-values.

## 1. Introduction

Networks are an incredibly versatile tool to model complex systems (Boccaletti, 2006). Besides the physical and natural sciences networks have become popular in the social and behavioral sciences (see e.g., Borsboom et al., 2011; Cramer et al., 2010; De Ron et al., 2019). One of the main areas of application is psychopathology (e.g., Cramer et al., 2016; Fried et al., 2016; Robinaugh et al., 2019), but networks have also been applied to personality research (Costantini et al., 2015), sleep (Blanken et al., 2020) and health psychology (Hevey, 2018).

Most networks are currently estimated with the least absolute shrinkage and selection operator (lasso), which shrinks all parameter estimates toward zero and sets very small estimates to exactly zero (e.g., Epskamp et al., 2018b; Epskamp & Fried, 2018; Haslbeck & Waldorp, 2015; 2020; van Borkulo et al., 2014). The lasso thereby does not only provide parameter estimates but also selects which edges are included in a network. This property makes this estimator very attractive, but it also has downsides. First, due to the shrinkage (bootstrapped) sampling distributions are biased and distorted and do not allow one to compute frequentist confidence intervals (CIs; Bühlmann & Meinshausen, 2014; Williams, 2021). However, CIs would be a useful and straightforward way to quantify the uncertainty about parameter estimates that is currently missing in frequentist network estimation. Second, the lasso has good properties (i.e., low false and high true positive rates) if its assumptions are met (e.g., Hastie et al., 2019). However, when those assumptions are not met, alternative methods might be preferable that have fewer and less strong assumptions, which may be more plausible in empirical situations, such as ridge regression (Bühlmann et al., 2013; Hoerl & Kennard, 1970) or best subset regression (Bertsimas et al., 2016; Brusco et al., 2022a; Hastie et al., 2017).

In this article, we investigate three methods to perform inference using the lasso, which allow us to obtain unbiased CIs and perform network inference with fewer assumptions than the lasso. Specifically, we will consider the multi-split method (Javanmard & Montanari, 2014; Meinshausen et al., 2009; Zhang & Zhang, 2014), the desparsified lasso (van de Geer et al., 2014), and a bootstrap version of the desparsified lasso (Dezeure et al., 2015). Although there are other methods to obtain correct inference with the lasso, such methods are either very similar to the ones we selected (Taylor & Tibshirani, 2018, extended results from Lee et al., 2016 and Lockhart et al., 2014), or they appear less appropriate considering the assumptions (Lu et al., 2017). We review the assumptions of the multi sample-split and desparsified based approaches, and we evaluate the performance of each of the methods and other popular methods in a simulation study. In the simulation, we recover Gaussian Graphical Models (GGMs), showing that the desparsified lasso performs best. Finally, we provide a fully reproducible R-tutorial on how to use the desparsified lasso to estimate a GGM on a dataset of post-traumatic stress disorder symptoms.

## 2. Gaussian graphical models

**Figure 1.**
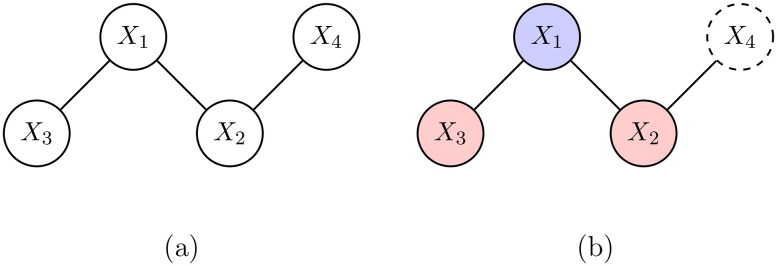
(a) Example of a network with four nodes 1, 2, 3 and 4 with associated variables X1, X2, X3, and X4. The edges X1−X2, X1−X3 and X2−X4 correspond to the conditional dependence statements, and the absence of edges correspond to conditional independence statements: X2⊥⊥X3|X1, X1⊥⊥X4|X2 and X3⊥⊥X4|X1,X2. (b) The variable X4 is not part of the neighborhood of X1, while X2 and X3 are part of the neighborhood of X1.

A Gaussian graphical model (GGM) is an undirected graph (network) where the nodes represent the variables and the edges represent conditional dependence, or, what is equivalent for Gaussian random variables, conditional correlation (Maathuis et al., 2018). The nodes in the network are labeled by integers 1,2,…,p, and this set of node labels is referred to as *V*. The edges of the network are denoted by (i,j) for nodes *i* and *j*. An example of a network is shown in Figure 1(a).

To each node *j*, we associate a random variable $X_{j},$ for j=1,2,…,p. The edges between the variables in the network indicate conditional independence: if there is no edge between node *i* and *j*, then variables Xi and Xj are independent given all other remaining variables Xk, which we write as Xi ⊥⊥ Xj∣Xk (Dawid, 1979). The combination of the network and a distribution over all random variables X1,X2,…,Xp is called a graphical model. When the multivariate distribution over all random variables is Gaussian (normal), we call the network together with the distribution a Gaussian Graphical Model (GGM). In Appendix A, we provide more details on the GGM, including a small example illustrating the relation between the partial covariance and conditional independence. Here, we suffice with a brief summary of the most relevant properties.

It turns out that whenever the correlation ρij between the variables Xi and Xj is ≠0, then there is a path of edges in the network between nodes *i* and *j* (Jones & West, 2005). However, we cannot determine what the path is by considering only correlations. We must investigate partial correlations (or partial covariances), i.e., correlations where all other variables are kept fixed (i.e., conditioned on). This way the influence of the other variables is removed, and the correlations between two residuals after regressing out all other variables is considered (Koller & Friedman, 2009). The partial covariance between Xi and Xj given all other remaining variables is denoted by Θij. Lauritzen (1996, Proposition 5.2) proved that if the partial covariance between variables Xi and Xj is 0, i.e., Θij=0, then the two variables are independent conditional on all other remaining variables X1,…,Xp without Xi,Xj. Because the partial correlation is a scaled version of the partial covariance, we may also determine whether the partial correlation is 0 to conclude that two variables are conditionally independent. Consequently, by knowing which two variables have 0 partial covariance (or 0 partial correlation), we know which variables should not be connected in the network. Each of the non-zero partial covariances between two variables will obtain an edge in the network.

## 3. Estimation of GGM with the lasso

To determine whether the partial covariance Θij is 0, we first need to estimate it from the data. It is convenient to determine the partial covariances nodewise, i.e., estimate the coefficients of the connected nodes for each node separately. This is often referred to as neighborhood or nodewise selection (Hastie et al., 2015). The complete network can be considered as a set of neighborhoods, where each neighborhood is the set of nodes that is connected to a particular node. Consider Figure 1(b), where node 1 is selected and the neighborhood consists of nodes 2 and 3, and not 4. If we obtain the neighborhoods for all nodes, then we can stitch these together and obtain the entire network. This has the advantage that we can use the linear regression framework. The regression of node *i* on the remaining nodes j≠i is

$$Xi=\sum_{j\neq i}Xj\beta \text{ij}+ei$$

where the sum is over all nodes *j* except *i* (which is now the dependent variable), βij are the regression coefficients, and ei is the residual of node *i*, which we assume to normally distributed with variance $\sigma_{e}^{2}$ and independent from other variables. We explicitly use the indices *ij* in the coefficient βij to make clear that it is about the relation between nodes *i* and *j* in the network. The reason we can use this regression framework is because of the relation between the coefficient βij and the partial covariance Θij, which is (Lauritzen, 1996; Waldorp & Marsman, 2022);

$$\beta \text{ij}=-\frac{\Theta \text{ij}}{\Theta \text{ii}}$$

So, if βij=0 then also Θij=0. In the example of Figure 1(b), we first select variable X1 as the dependent variable and predict it by X2, X3 and X4. If β14=0, then we do not draw an edge between X1 and X4. If β12 and β13 are non-zero, then the edges between X1 and X2 between X1 and X3 are drawn.

Estimation is often performed by using a penalized form like the least absolute shrinkage and selection operator (lasso, Tibshirani, 1996). For regression with the lasso, the coefficients βij for each node *i* are obtained by a combination of least squares and a penalty, the sum of absolute values of the coefficients weighted by a penality parameter (see Appendix B for details). A lasso estimate for βij is denoted by $\hat{\beta }\text{ij}.$ The lasso has the advantage that by estimating the coefficients βij, the coefficients are also selected because some of the estimated coefficients will be exactly 0 (Bühlmann & van de Geer, 2011; Tibshirani, 1996). Additionally, the lasso has the advantage that it decreases prediction error (i.e., the error between prediction using the estimate and the true parameter, Hastie et al., 2015). The lasso penalty parameter, usually denoted by λ, needs to be determined, and so, an estimate depends on the value λ chosen. Specifically, larger values of λ result in stronger regularization. Often the value for λ is obtained by using cross-validation (see Appendix B).

It has been shown that the lasso obtains few false positives and many true positives (Wainwright, 2009), but this requires several assumptions. The assumptions are

- the true parameter vector is *sparse* i.e., the number of non-zero coefficients in the true parameter vector is at most *s*, which is much smaller than the number of observations *n*,
- the variables are not too highly correlated i.e., the variables are *not collinear*,
- the correlation between connected and not connected variables cannot be 1 (*incoherence*), and
- non-zero coefficients must be at least larger than some small value βmin.

Especially the last assumption about small values in the true parameter is problematic because with the lasso we have to assume that these do not exist (Dezeure et al., 2015). We review the assumptions in more detail in Appendix B.

## 4. Inference for GGM estimated with the lasso

We are interested in obtaining *p*-values or confidence intervals after we have selected a particular model with the lasso. Those are useful because they are a natural way to quantify uncertainty about parameter estimates. However, we will also see that selecting edges with *p*-values can be advantageous if the assumptions of the lasso are violated. In what follows we focus on *p*-values but we provide details on obtaining confidence intervals in Appendix G.

We first discuss the regular (non-penalized) situation to obtain *p*-values. An estimate ${\hat{\beta }}_{\text{ij}}^{\text{LS}}$ for the parameter βij obtained with least squares (see Appendix B) has a normal distribution (if the errors are normally distributed, which we assume here). And so we can use the standard error $\text{se}({\hat{\beta }}_{\text{ij}}^{\text{LS}})$ (the standard deviation of the distribution of ${\hat{\beta }}_{\text{ij}}^{\text{LS}}$) to obtain a *p*-value. The corresponding hypotheses for each parameter *j* for the *i*th regression are the null and alternative hypotheses, respectively

(1) H0,ij:βij=0 versus Ha,ij:βij≠0

For each of these hypotheses, we obtain a *p*-value pij (see Appendix C). Since a network has many edges to be tested, the family wise error rate increases with the number of tests, i.e., the problem of multiple testing arises. We use the Bonferroni-Holm procedure (Hochberg & Tamhane, 1987) for multiple testing correction (see Appendix C for details on calculating corrected *p*-values with the Bonferroni-Holm procedure).

The problem with inference using the lasso is that, in general, the distribution of the parameter estimates $\hat{\beta }\text{ij}$ obtained with the lasso is rather difficult (Bühlmann et al., 2013; Knight & Fu, 2000; Pötscher & Leeb, 2009). The issue is that because lasso estimates can be exactly zero, the limiting (as n→∞) distribution is discontinuous and may have probability one at zero whenever the true parameter value is zero (Dezeure et al., 2015; Knight & Fu, 2000). As a consequence, standard methods for calculating standard errors are not possible (Tibshirani, 1996) and also standard bootstrap methods do not work (Chatterjee & Lahiri, 2011; Knight & Fu, 2000).

**Figure 2.**
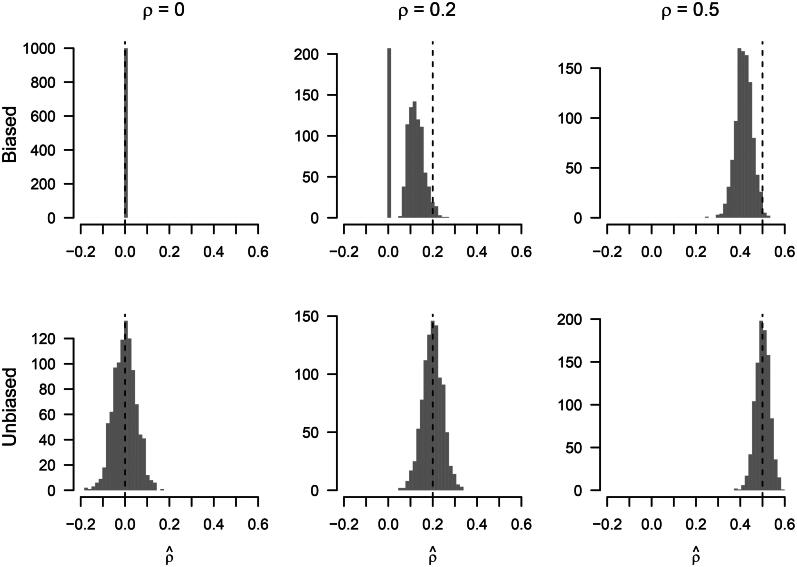
Top panel: bootstrapped sampling distributions of lasso estimates of partial correlations ρ=0,0.2,0.5. Bottom panel: the corresponding bootstrapped sampling distributions based on unbiased least squares estimates. The dashed vertical line indicates the true parameter.

We illustrate the issue in Figure 2 with computing confidence intervals based on bootstrap samples from lasso estimates. Here, we considered a GGM with p=20 variables and nine nonzero partial correlations ρ=0.1 up to ρ=0.9. In the top panel of Figure 2, we display the sampling distributions obtained from the lasso estimates with n=500 and B=1000 bootstrap samples for partial correlations of sizes ρ=0, 0.2 and 0.5. Clearly, the sampling distributions are not normally distributed like the ones of the unbiased OLS estimator in the bottom row. For example, for ρ=0, all estimates are zero and for ρ=0.2, a large number of estimates are zero, then there is a gap, and then the rest of the estimates are distributed roughly around 0.1.

A central issue with these biased sampling distributions is that their quantiles do not correspond to frequentist confidence intervals, which have the property that they include the true parameter with probability 1−α (i.e., coverage). This probability can be approximated by computing many CIs, but from the top panel of Figure 2, we can already see that the coverage will be much lower than desired. This is especially a problem for true parameters that are not close to zero. For example, the 95% quantiles of the biased sampling distribution of ρ=0.5 are 0.341 and 0.488, which does not include the true parameter 0.5. The lower panel shows sampling distributions of the unbiased least squares estimates (see Appendix G). We see that the sampling distributions are roughly centered around the true estimates, which already suggests a higher coverage rate, and of course for unbiased sampling distributions the expected coverage is exactly 1−α (in the case of a correct GGM). Williams (2021) provides a simulation study that maps out the coverage of confidence intervals based on standard lasso estimates (without thresholding) in the regression and GGM context in more detail.

If we are interested in unbiased sampling distributions, why do we not simply perform inference with the standard OLS estimates? There are two reasons for this. First, we could have approximately the same number of nodes as the number of observations (e.g., 20 nodes and 50 observations). Such cases are found in situations of clinical populations where subjects are not easily obtained. This means that the number of observations is too low for reliable OLS estimates. Second, if the weaker assumptions of the methods we will consider below are satisfied, we would expect them to outperform inference on OLS estimates. In the simulation study in Section 5, we will see that this is indeed the case.

In the remainder of this section, we discuss the three methods to perform inference with the lasso. In the following section, we evaluate the performance of all methods in a simulation study.

### 4.1. Multi sample-split of the lasso

To circumvent the problem with the biased distribution of the lasso parameter estimates, the multi sample-split method uses a two-step procedure where in the first step the model is selected using the lasso, and in the second step, the *p*-values are obtained using a regular non-penalized method. The *p*-values obtained in the second step are obtained using an independent sample (the sample has been split) and using regular estimation (not lasso) and so can be interpreted in the normal way.

In the single split procedure, the sample is split in two subsets of the original sample, such that N1 contains a random subset of the original sample and N2 contains the remaining subset of the original sample, so that N1 and N2 do not share any element and together N1 and N2 are the original sample. The number of elements in N1, referred to as n1 is about half of the original sample, i.e., N1 is of size n1 and is approximately the same as n2, the size of the second half N2. Then the first half N1 is used to determine which edges should be included in the network. The second half of the sample N2 is then used to obtain regular *p*-values using only those edges in $\hat{S}1$ that have been selected using the first half of the sample, and no penalty like the lasso is required to obtain the *p*-values with the second half of the sample. It is possible to obtain *p*-values only if the selected number of edges per node is less than the size of the second sample. The fact that each node is allowed up to n2 edges is so that the coefficients in the regression are identified.

An advantage of the single sample-split method is that the selection of relevant variables using the first half of the sample is often much smaller than the number of nodes *p* (i.e., |S1| is much smaller than *p*), so that the *p*-values can be determined with more accuracy (Meinshausen et al., 2009). Another advantage is that obtaining corrected *p*-values for multiple testing is less conservative (i.e., will lead to smaller *p*-values) with the single sample-split because the correction factor is the number of selected variables in $\hat{S}1,$ which is smaller than p−1. For example, in the Bonferroni-Holm procedure (Hochberg & Tamhane, 1987, see also Appendix C), the *p*-values pij are sorted in ascending order, and denoted by p(ij). Let *m* be the number of tests to be performed, here p(p−1), all edges twice. Then, the Bonferroni-Holm correction for the uncorrected *p*-value pij will be for the regular and single sample-split method, respectively,

$$p_{\left(\text{ij}\right)}^{\text{BH}}=p\left(\text{ij}\right)(m-i-j)\;\;\text{versus}\;\;p_{\left(\text{ij}\right)}^{\text{BH}}=p\left(\text{ij}\right)(|\hat{S}1|-i-j)$$

where $\left|\hat{S}1\right|$ denotes the number of selected regressors in step 1.

For example, if the network has p=20 nodes then we need to test for a total of m=380 edges. Suppose that $|\hat{S}1|=12$ edges are retained from the first half of the sample (i.e., we selected 12 edges). Using the above equations, we obtain the Bonferroni-Holm correction factor, for the first *p*-value p(11), which is 378 for the regular method but only 10 for the single sample-split method. Consequently, without the sample-split selection in the first stage, we would be multiplying the first *p*-value with 378, while with the multi-split method this factor is reduced to 10 in this example. So, if the assumption is that the network is sparse (does not have too many edges), then this method obtains great gains in the probability to correctly detect edges.

The algorithm for the single sample-split is as follows.

1. Split the sample in two halves, N1 and N2, such that each has about the same size n1≈n2.
2. Apply lasso estimation using only N1, and put the selected edges in $\hat{S}1.$
3. Set the *p*-values for the edges that were not selected to 1, and using N2 compute the *p*-values only for the edges in $\hat{S}1$
4. Correct the *p*-values using the number of estimated edges in $\hat{S}1$

The single sample-split method has one great disadvantage, and that is that the way the sample split turns out is random. That means that another split could lead to different *p*-values (Meinshausen et al., 2009). This is undesirable because for one split a *p*-value for an edge could be small, while for another split for the same edge, the *p*-value could be large. To avoid such undesirable results, Meinshausen et al. (2009) came up with the idea to run multiple splits and then combine these splits in a single *p*-value.

In the multi sample-split procedure, the single sample-split is performed *K* times, so that we obtain for each *p*-value $p_{\text{ij}}^{k},$ *K* versions from a different half-split. This gives a distribution of *p*-values for each $p_{\text{ij}}^{k}$ and a criterion to aggregate these *p*-values is then based on a quantile from the empirical distribution function (Bühlmann et al., 2014; Meinshausen et al., 2009). Intuitively, the criterion is based on finding a quantile such that a correction factor is obtained that results in an approximately correct false positive rate (see Appendix D for more details). The main advantage of the multi sample-split is that no single sample-split determines the *p*-values and, hence, the result is approximately reproducible.

The assumptions for the multi sample-split method are all four assumptions (*a*)-(*d*) of the lasso in Section 3. The assumptions lead to the so-called *screening property*, which is well-known in the literature on high-dimensional statistics (see e.g., Bühlmann et al., 2013). It refers to the idea that we may obtain some spurious connections but will not miss-out on any relevant edges. It is clear that for the two-step procedure of the multi sample-split this is important: If in the first step some of the connections are missing, then the correct graph will never be obtained.

### 4.2. Desparsified lasso

The desparsified lasso makes inference with the lasso possible by modifying the original lasso estimate, adding a term depending on the residuals (Javanmard & Montanari, 2014; van de Geer et al., 2014). The desparsified lasso estimate no longer has exact 0 values in its estimate and hence is desparsified. The term being added is to remove the bias incurred by using the lasso penalty, and hence, the method is also called the debiased lasso (Bühlmann et al., 2014; Waldorp, 2014; Williams, 2020). The upshot of desparsifying the lasso is that the distribution of the estimate is approximately Gaussian and hence standard errors can be computed, which can be used to calculate *p*-values. The details of the desparsified lasso are given in Appendix E. The assumptions for the desparsified lasso are sparsity (*a*) and no collinearity (*b*) from Section 3.

### 4.3. Bootstrap of the desparsified lasso

Generally, there are two types of bootstrap in regression: the naive or pairs bootstrap and the residual bootstrap (called resampling cases and model based resampling, respectively, in Davison & Hinkley, 1997, Chapter 6). In the pairs (or non-parametric) bootstrap each pair of dependent and independent variables is resampled, with the underlying assumption that these observations have been obtained from a multivariate distribution. In the residual (or parametric) bootstrap, the independent variables are resampled and are then used to create residuals (together with (a model for) the mean). These residuals are, together with the model, used to create resampled versions of the dependent variable. We focus here on the pairs bootstrap, because this fits more with the idea of having obtained data from a network, which clearly assumes an underlying multivariate distribution.

To obtain a bootstrap *p*-value, an empirical distribution is created by resampling from the original observations with replacement such that for each resample we obtain an estimate. With these estimates, we can create an empirical distribution from which we can obtain a *p*-value. To estimate the parameters, we use the desparsified lasso because in that case we are assured that an approximate normal distribution underlies the estimates. The pairs bootstrap for a network with *n* identical observations of all variables is as follows.

1. Sample randomly from the observations x1i,x2i,…,xni, with replacement, for all i=1,2,…,p simultaneously. Call these resampled observations $x_{1i}^{⋆},x_{2i}^{⋆},\ldots ,x_{\text{ni}}^{⋆}.$
2. Perform nodewise regressions using the desparsified lasso to the resampled data $x_{1i}^{⋆},x_{2i}^{⋆},\ldots ,x_{\text{ni}}^{⋆}$ for all *i* (Section 3) to obtain parameters ${\hat{\beta }}_{\text{ij}}^{⋆,d}.$
3. Repeat steps (1) and (2) *K* times.
4. Obtain a *p*-value from the empirical distribution of the *K* bootstrap versions of ${\hat{\beta }}_{\text{ij}}^{⋆,d}.$

The assumptions of the bootstrap are those required for the desparsified lasso estimates ${\hat{\beta }}_{\text{ij}}^{d}$ to have a proper distribution. The assumptions of sparsity (*a*) and no collinearity (*b*) imply that the estimates have a proper distribution. To obtain accurate *p*-values, the number of bootstrap resamples should be in the order of at least 500 (Davison & Hinkley, 1997, Chapter 2).

### 4.4. A brief comparison of assumptions

Each of the methods depends on their assumptions, just like the selection of the lasso. In the previous section, assumptions (*a*)–(*d*) were mentioned for the lasso to be able to select the correct network. Each of these assumptions is necessary for correct identification of the network edges (see Wainwright, 2009, for an excellent discussion on arguments of these assumptions). Except for collinearity, assumption (*b*), none of the assumptions can be tested empirically. And so, fewer assumptions about the nature of the data generating process are better. Table 1 provides an overview of the assumptions of each method. It is clear that from the perspective of the number of unwarranted assumptions that the desparsified lasso and the bootstrap based on the desparsified lasso should be preferred.

For reference, we have also included the ordinary least squares (OLS) and graphical lasso (glasso) estimators in Table 1. OLS refers to estimation of the edge parameters without constraints (see Appendix B). And glasso refers to estimation of the edge parameters by applying the lasso columnwise to the covariance matrix (Friedman et al., 2008a). This is a relatively popular technique, featuring in several R packages like *qgraph* and *huge*.

## 5. Evaluating performance of all methods via simulations

To determine which inference method performs best in determining the structure of GGMs, we evaluated randomly generated networks with 20 nodes and different numbers of observations, i.e., 50, 100, 200, 500, 800 and 1000. Additionally, we varied the number of edges in the network to determine the effect of violating sparsity, and we varied the size of the coefficients of the edges to determine the effect of violating the beta-min assumption.

The edges in the network are randomly placed between two nodes with probability of connection pe, which we set to either pe=0.2 or pe=0.4. Using the common approximation of sparsity $s=\sqrt{n/\;\text{log}\;(p)}$ (e.g., Bühlmann et al., 2014), we can determine to what extent the sparsity assumption is met on the nodewise level in each simulation condition. For example, for p=20 variables and n=50 only $\sqrt{50/\;\text{log}\;(20)}\approx 4$ edges are allowed in the neighborhood of variable *i* in order for sparsity to be satisfied. For the edge probabilities pe=0.2 or pe=0.4, this implies that the sparsity assumption will on average be violated for 9 and 20 nodes, respectively (for the deails of these calculations see Appendix B). Table 2 displays for each simulation condition for how many nodes the sparsity assumption is violated.

We learn from the comparison between the sparsity and the number of connections for the nodes that when n=50 almost half of the nodes violate the sparsity assumption (*a*) when pe=0.2 and that all nodes violate the sparsity assumption when pe=0.4. When n=500 the sparsity assumption is not violated in either the network with pe=0.2 or 0.4. These two values of the probability of connection will show the effect of sparsity on the performance of each of the methods.

With this network, we generated partial covariances with coefficients Θij that were randomly drawn from the uniform distribution with values between 0.1 and 0.8. Using the heuristic of the beta-min value $d=\sqrt{\;\text{log}\;(p)/n}$ (e.g., Bühlmann et al., 2014), we find that for n=500 observations all values are above the heuristic beta-min value of approximately 0.08, while for n=50 the beta-min value is approximately 0.25, indicating that between 70% and 80% of the coefficients are above the beta-min threshold (see Table 2). So, the differences in coefficients allows us to determine the effect of violating the beta-min assumption. With these partial covariances we then generated multivariate normal data (see Appendix H for more details).

We obtained the *p*-values with one of the three methods: the multi sample-split, the desparsified lasso and the bootstrap (with the desparsified lasso). For each of the three methods, we used the Bonferroni-Holm correction to obtain corrected *p*-values (see Appendix C) with family wise error rate 0.05. The penalty parameter λ was obtained with 10-fold cross-validation. In the nodewise regression-based methods, we combined estimates with the *and*-rule.

In order to evaluate the impact of satisfying different assumptions, we extended the simulation with the lasso with and without additional thresholding (Loh & Wainwright, 2012), where we select the nodewise regularization parameter λi with 10-fold cross-validation (Haslbeck & Waldorp, 2020) and a procedure estimating the GGM using standard OLS regression and significance tests with Bonferroni-Holm correction. In addition, we considered the graphical lasso (Friedman et al., 2008b) where we select the global regularization parameter λ with the Extended Bayesian Information Criterion (EBIC; Foygel & Drton, 2010), due to its popularity in applied research (Epskamp et al., 2018a). More details of the settings for the simulation can be found in Appendix H.

We use as measures of performance

$$\text{sensitivity}:\frac{\#\{\text{edge significant }\text{ and edge present}\}}{\#\{\text{edge present}\}}$$

and

$$\text{precision}:\frac{\#\{\text{edge significant }\text{and edge present}\}}{\#\{\text{edge significant}\}}$$

where #{a} denotes the number of objects with property *a*. Sensitivity is the same as the true positive rate or recall and measures the proportion of edges correctly identified. Precision is the same as the positive predictive value. Equivalently, precision counts the number of true positives with respect to the number of true and false positives. It, therefore, gives an indication of whether any of the edges that are deemed significant could be false positives. Both measures of accuracy have a value close to 1 to indicate that the method works well. We also consider specificity (#{edge not significant *and* edge absent}/#{edge absent}), shown in Appendix I.

To determine whether the confidence intervals are accurately estimated, we considered the coverage rate. By investigating the coverage rate, we can determine whether with the specified probability of 1−α, where we choose α=0.05, the true parameter is in the confidence interval with probability at least 0.95.

The code to fully reproduce the simulation study and the results below can be found at https://github.com/jmbh/networkinference_reparchive.

### 5.1. Results

**Figure 3.**
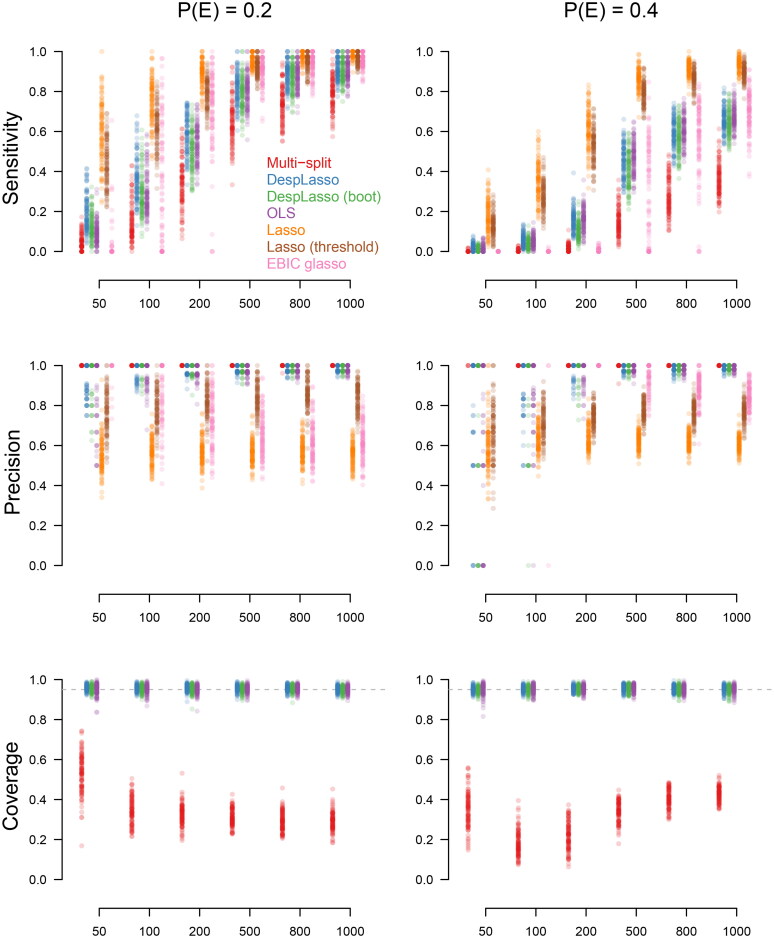
Sensitivity (first row), precision (second row), and coverage (third row) for edge probabilities 0.2 (left column) and 0.4 (right column), as a function of sample size (x-axis). The dashed horizontal line at 0.95 in the last row indicates the desired coverage based on the chosen threshold α=0.05.

Figure 3 shows the sensitivity (first row) and precision (second row) for the conditions with edge probabilities pe=0.2 (left column) and pe=0.4 (right column) for the seven considered estimation methods. With a relatively low number of true edges (pe=0.2), the ordinary least squares (OLS) and desparsified lasso (Desplasso) both have reasonable sensitivity and high precision. The desparsified lasso has slightly better sensitivity and precision than OLS, especially when the sample size is relatively small. Additionally, the desparsified lasso has less variation in precision than OLS, again especially for the smaller sample sizes. These results are mostly confirmed when considering true absent edges (specificity, see Appendix I). This is to be expected because with OLS smaller coefficients tend to be selected more easily, whereas they are biased toward 0, and hence not selected, with the desparsified lasso.

The bootstrap desparsified lasso is also quite accurate but has slightly lower sensitivity and precision than the desparsified lasso. The multi sample-split has excellent precision but, as a consequence of this excellent precision, has lower sensitivity than the desparsified lasso. The regular lasso has high sensitivity but poor precision. Note that the lasso has a much higher variation in both sensitivity and precision than any of the other methods, which makes it difficult to ascertain in a single dataset if sensitivity is high or not. The lasso will therefore, without thresholding, lead to a high false positive rate. This is to be expected, since the guarantees for the lasso are only obtained when the beta-min assumption is satisfied and, hence, requires thresholding (Wainwright, 2009). We performed additional simulations with the lasso and thresholding showing that precision can be much improved upon compared to the lasso without thresholding (lasso thresholded in Figure 3). However, applying the Bonferroni-Holm correction to the desparsified lasso has both higher sensitivity and precision than the thresholded lasso. The EBIC glasso has high sensitivity but its precision is relatively low, which drops further as the sample size increases.

With a larger number of edges in the network (pe=0.4, bottom row Figure 3), the sensitivity of all methods drops significantly. But even when the sample size is n≥500, then the number of edges to each node is about 12 (see Table 2) and no nodes in the graph violate the sparsity assumption. But it is clear that both unregularised (i.e., OLS) and the desparsified lasso estimates suffer from the number of edges to be selected. The performance of OLS and the desparsified lasso is similar, also in the case of sample size n=800 or n=1000. Because in these cases, there are no sparsity or beta-min violations, the drop in performance can be attributed to the large number of parameters to be estimated in the model compared to the number of observations. Precision is quite similar for OLS and the desparsified lasso to the case where sparsity holds. The only difference in precision when sparsity does not hold is that for OLS there are some cases that have nearly 0 precision, so none of the selected edges are correct. The drop in performance of the multi-split method shows that splitting the data and then first selecting and then estimating leads to much lower sensitivity. But precision remains high for the multi-split method. The EBIC glasso shows comparable sensitivity as OLS and the desparsified lasso, but the precision of the EBIC glasso is somewhat lower, especially for larger samples, making it a less attractive option.

The bottom row in Figure 3 shows the coverage of the confidence intervals. We see that nearly all types of confidence intervals are accurate, and especially the two versions of the desparsified lasso and OLS have coverage centered around the desired value of 0.95. In contrast, the multi-split method has low coverage. As shown in Appendix I (two lower rows), we notice that this poor performance of the multi-split method is mainly because this method does not cover true absent edges well. The multi-split method was designed for hypothesis testing, and as a result, it does not perform well on coverage of confidence intervals.

In conclusion, the best balance between sensitivity and precision is obtained with OLS and the desparsified lasso (also bootstrapped) with a bit higher precision (lower false positive rate) for the desparsified lasso, especially at lower sample sizes. This leads to a recommendation for the desparsified lasso in general, but more so in cases where the sample size is similar (in order) to the number of expected edges of the nodes. For instance, with 20 nodes and 100 observations we would recommend using the desparsified lasso.

## 6. Tutorial: Inference on regularized networks with *inet*

Here, we show how to use the R-package *inet* to perform inference on the edges of Gaussian graphical models and how to inspect the confidence intervals associated with each edge. We use cross-sectional data on 17 PTSD symptoms of N=344 individuals from the study of McNally et al. (2015). These data are loaded automatically with the *inet* package, which is available on the The Comprehensive R Archive Network (CRAN).

We use the desparsified lasso to perform inference on the edges of a Gaussian graphical model. To do so we use the function lasso_dsp and provide the data and the desired significance level as input:

library(inet) out <- lasso_dsp(data = ptsd_data, ci. level = 0.95)

**Figure 4.**
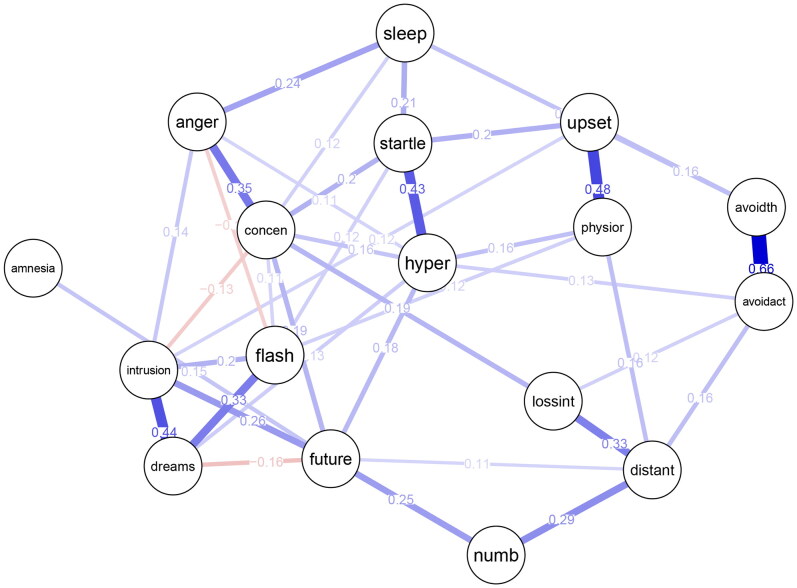
Significant partial correlations in the PTSD data; blue/red edges correspond to positive/negative partial correlations; the width of edges is a function of the absolute value of the associated parameter.

The output object consists of the point estimates (out$est), the significant point estimates (out$est.signf), a matrix indicating whether each estimate is significant (out$signf), and the lower and upper bounds of the confidence interval (out$ci.lower and out$ci.upper). Next, we create a network visualization of the significant point estimates in out$est.signf using the R-package *qgraph*: (see Figure 4)

library(qgraph) qgraph(out$est.signf, labels = colnames(ptsd_data), layout = "spring", edge.labels = TRUE, theme = "colorblind")

Next, we inspect the confidence intervals. Instead of inspecting them manually, they can be plotted using the generic plot() function. Here, we choose to order the parameters by decreasing absolute value of the point estimate (order = TRUE) and to only display the first 50 parameters in that ordering:

plot(object = out, labels = colnames(ptsd_data), order = TRUE, subset = 1:50)

**Figure 5.**
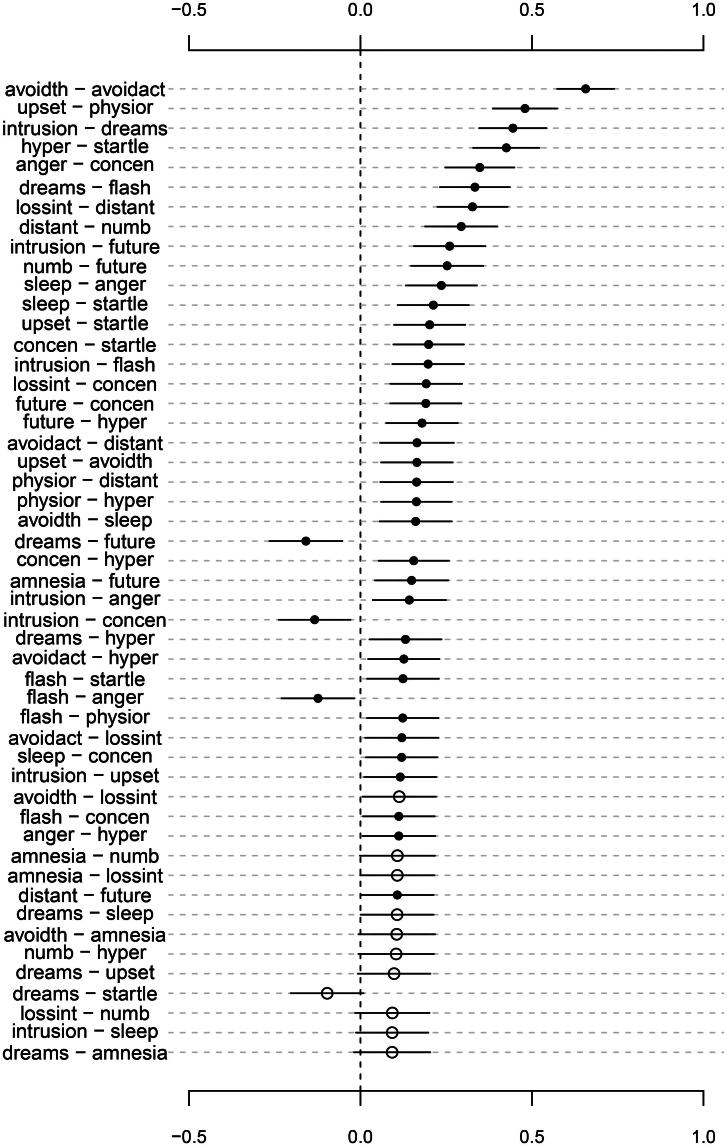
The point estimates and confidence intervals of the 50 partial correlations with the largest point estimate. Hollow points represent estimates whose CIs overlap with zero.

The resulting plot is shown in Figure 5.

## 7. Discussion

We have reviewed and evaluated the performance of three different methods to obtain *p*-values (and confidence intervals) for network estimates: the multi sample-split, the desparsified lasso and the bootstrap based on the desparsified lasso. For comparison, we also included the lasso (with and without thresholding), the glasso with the extended BIC for the selection of the penalty parameter, and ordinary least squares. The desparsified lasso methods have higher sensitivity than OLS at small samples and similar sensitivity at large samples. Additionally, the desparsified lasso methods have less variation in precision than OLS. The reason is that the assumption of sparsity helps to decrease small coefficients (edges) to 0 that are most likely not true edges. In this evaluation we considered the effects of the different assumptions: (*a*) sparsity, (*b*) non-collinearity, (*c*) incoherence, and (*d*) beta-min. By increasing sample size, we decreased the level of permitted sparsity and the beta-min threshold. Considering the balance between sensitivity and precision from the simulations and having few assumptions, the best method is obtained with the desparsified lasso.

Here, we focused on the lasso for estimation and then desired to obtain correct inference from the lasso estimate. However, as mentioned in the Introduction, if the assumptions of the lasso (*a*)–(*d*) are unrealistic for the application at hand, then it might be prudent to consider alternatives. An excellent alternative is ridge regression (Bühlmann et al., 2013; Hoerl & Kennard, 1970). In ridge regression, the squares of the parameters are used in the penalty, which makes it easier to obtain correct inference (but does not select coefficients like the lasso). Another alternative is the recently suggested best subset regression, where the penalty is simply the number of allowed non-zero coefficients (Bertsimas et al., 2016 and Brusco et al., 2022a, but see also Hastie et al., 2017, for a comparison with the lasso). The best subset selection, like the lasso, selects the number of non-zero coefficients, but inference is also somewhat involved.

Here, we investigated three options for inference when using the lasso: the multi sample-split, the desparsified lasso and a bootstrap version of the desparsified lasso. We discuss each in turn.

The multi sample-split is an intuitive and straightforward method and, therefore, has great appeal. Compared to other methods, however, it has low sensitivity and, it breaks down rather dramatically when the number of true edges is large compared to the number of observations (i.e., the solution is not sparse). This is because the multi-split method halves the data (repeatedly) to select edges and then estimates the *p*-values. This halving of the sample size has a significant effect on the sensitivity of the multi-split method compared to all other methods. Also, the assumptions required for the multi sample-split are the four assumptions (*a*)–(*d*), precisely those that are required for accurate network estimation with the lasso. Both the incoherence (*c*) and beta-min (*d*) assumptions are formidable, in that they cannot be tested in practice, and, they assume particular properties of the underlying distribution that can never be guaranteed. Hence, because of poorer performance compared to other methods and the full set of assumptions required, the multi-split method is not recommended in general.

Using the desparsified lasso means that we do not require the assumptions (*c*) about incoherence and (*d*) about the smallest detectible coefficient (beta-min). In contrast, the lasso (normal version) does require these two assumptions for correct network selection. The simulations showed that the desparsified lasso has excellent performance when sparsity holds. It performs similarly to unregularised estimation and selection (OLS), and outperforms OLS for small sample sizes. This implies that in any case, assuming sparsity or not, the desparsified lasso appears to be an attractive method for inference.

The bootstrap based on the desparsified lasso shows similar performance as the desparsified lasso. This is similar to the situation without the lasso, where the bootstrap in standard situations (like Gaussian random variables) performs similarly to the parametric versions. In terms of assumptions, these are the same as the desparsified lasso. The theory of the desparsified lasso mostly carries over to generalized linear models (van de Geer et al., 2014, Theorem 3.3). Because the loss function is not always convex, more assumptions are required to guarantee the high recall and precision. These assumptions may affect finite sample behavior differently than for the GGM. Statements about finite sample performance with generalized linear models requires further investigation, but for binary random variables (Ising model), some results have been obtained (Brusco et al., 2022b; Waldorp et al., 2019).

In conclusion, the desparsified lasso and the bootstrap based on the desparsified lasso seem to be the best option for estimating GGMs. In cases where obtaining *p*-values or confidence intervals is more problematic, the bootstrap based on the desparsified lasso seems to be best.

## Figures and Tables

**Figure I.1.**
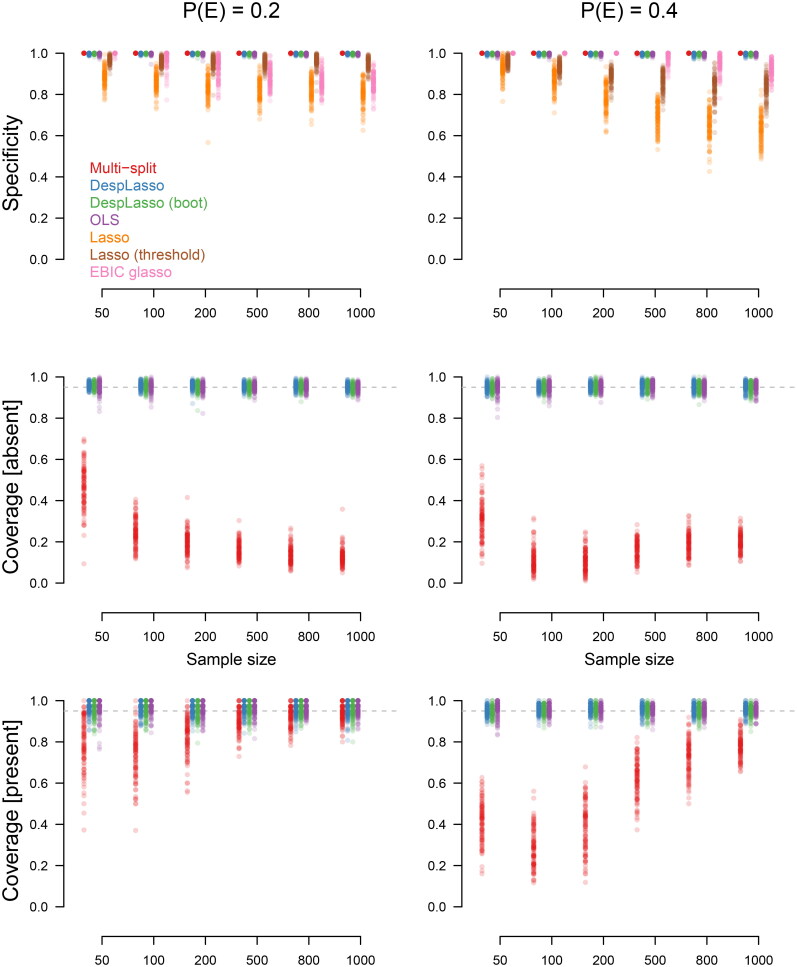
Specificity (first row), coverage for absent edges (second row), and coverage for present edges (third row) for edge probabilities 0.2 (left column) and 0.4 (right column), as a function of sample size (x-axis).

## Article information

**Conflict of interest disclosures:** The authors report there are no competing interests to declare.

**Ethical principles:** The authors affirm having followed professional ethical guidelines in preparing this work. These guidelines include obtaining informed consent from human participants, maintaining ethical treatment and respect for the rights of human or animal participants, and ensuring the privacy of participants and their data, such as ensuring that individual participants cannot be identified in reported results or from publicly available original or archival data.

**Funding:** This work was supported by Gravity project ‘New Science of Mental Disorders’, the Dutch Research Council and the Dutch Ministry of Education, Culture and Science (NWO), grant number 024.004.016.

**Role of the funders/sponsors:** None of the funders or sponsors of this research had any role in the design and conduct of the study; collection, management, analysis, and interpretation of data; preparation, review, or approval of the manuscript; or decision to submit the manuscript for publication.

**Acknowledgements:** We would like to thank the editors and reviewers for their constructive comments on our manuscript. The ideas and opinions expressed herein are those of the authors alone, and endorsement by the University of Amsterdam is not implied.

## Appendix

### A. Gaussian Graphical Models

The Gaussian graphical model (GGM) refers to a network with nodes V={1,2,…,p} associated with the random variables X1,X2,…,Xp, distributed according to a multivariate Gaussian distribution. The edges between nodes *i* and *j* in *V* of the network, denoted by (i,j), are associated with the partial covariances, which we explain below. Two variables are independent if the joint distribution f(x1,x2) can be written as the product of marginal distributions f(x1)f(x2); this is denoted by X1 ⊥⊥ X2. The variables Xi and Xj are conditionally independent given an other variable Xm when the joint probability f(xi,xj,xm) can be written as f(xi∣xm)f(xj∣xm); this is denoted by Xi ⊥⊥ Xj∣Xm.

The GGM is completely determined by the mean vector and covariance matrix.

$$\mu =(\mu 1,\mu 2,\ldots ,\mu p)\;\;\text{and}\;\;\Sigma =\left(\begin{array}{cccc} \Sigma 11 & \Sigma 12 & \cdots & \Sigma 1p\\ \Sigma 21 & \Sigma 22 & & \vdots \\ \vdots & & \ddots & \\ \Sigma p1 & \cdots & & \Sigma \text{pp} \end{array}\right)$$

The inverse of the covariance matrix, denoted by $\Sigma^{-1}=\Theta ,$ refers to the relation ΣΘ=ΘΣ=Ip, where Ip is the identity matrix with all diagonal elements 1 and all off-diagonal elements 0 (see, e.g., Schott, 1997). For the identity matrix Ip, we have that IpA=AIp=A, for any p×p matrix *A*. This idea is similar to real numbers in R where for any a∈R there exists a b∈R such that ab=ba=1, and we call $b=a^{-1}.$

With the inverse of the covariance matrix $\Sigma^{-1}=\Theta ,$ the Gaussian (normal) multivariate distribution over X1,X2,…,Xp is (Bilodeau & Brenner, 1999, Chapter 5)

$$f\mu ,\Sigma (x)=\frac{1}{{\left(\sqrt{2\pi \text{ det }(\Sigma )}\right)}^{p}}\text{exp}\;\left(-\frac{1}{2}{\left(x-\mu \right)}^{⊤}\Theta (x-\mu )\right)$$

where det(Σ) is the determinant of Σ (see, e.g., Schott, 1997). A component Θij can be characterized by the correlation between the residuals $ei=yi-x_{-\text{ij}}^{⊤}\beta -\text{km}$ and $ej=yj-x_{-\text{ij}}^{⊤}\beta -\text{ij},$ where the subscript −ij refers to the *i*th and *j*th elements being left out. The correlation between the residuals ei and ej makes clear the idea that the partial correlation concerns the remaining correlation after all other variables (except *k* and *m*) have been removed.

It can be seen from the distribution function fμ,Σ above that the 0s in the partial covariance matrix Θ play a central role in a GGM. In fact, Lauritzen (1996, Proposition 5.2) showed that if the partial covariance Θij=0 then Xi and Xj are independent conditional on all other remaining variables, i.e.,

$$\underset{\text{partial covariance}=0}{\underbrace{\Theta \text{ij}=0}}\;\;\Leftrightarrow \;\;\underset{\text{conditional independence}}{\underbrace{Xi⊥⊥Xj|X1,\ldots ,Xp-(Xi,Xj)}}$$

This means that if Θij=0, then the distribution function can be written in product form, implying conditional independence.

To illustrate the relation between partial correlation and dependence for random variables with a multivariate Gaussian distribution, we provide a small example. Let x=(x1,x2) so that we have two variables, each assumed to have mean μ1=μ2=0. Suppose, furthermore, that the covariance and inverse (partial) covariance matrices are

$$\Sigma =\left(\begin{array}{cc} \frac{1}{2} & 0\\ 0 & \frac{1}{2} \end{array}\right)\;\;\text{and}\;\;\Theta =\left(\begin{array}{cc} 2 & 0\\ 0 & 2 \end{array}\right)$$

We immediately see that there is no connection between the two nodes because Θ12=0. In this case, there is no path between nodes 1 and 2 (there are no other nodes) and hence, the correlation is 0 (since Σ12=0). The determinant of Σ is $\text{det}(\Sigma )=\frac{1}{2}\cdot \frac{1}{2}=\frac{1}{4}.$ Plugging this in the multivariate Gaussian distribution above, we obtain

$$f\Sigma (x1,x2)=\frac{1}{2\pi \frac{1}{4}}\text{exp}\;\left(-\frac{1}{2}(2x_{1}^{2}+2x_{2}^{2})\right)$$

where we left out μ in fΣ because we assumed μ=0. We can equivalently write this as

$$f\Sigma (x1,x2)=\frac{1}{\sqrt{2\pi \frac{1}{2}}}\text{exp}\;\left(-\frac{1}{2}\frac{x_{1}^{2}}{1/2}\right)\cdot \frac{1}{\sqrt{2\pi \frac{1}{2}}}\text{exp}\;\left(-\frac{1}{2}\frac{x_{2}^{2}}{1/2}\right)=f\frac{1}{2}(x1)f\frac{1}{2}(x2)$$

We then see that the multivariate distribution can be written as the product of the individual distributions. Hence, assuming that the partial covariances (or partial correlations) are 0, leads to the variables being independent when the multivariate distribution is Gaussian.

Because correlations are easier to interpret, the partial correlations are often used in GGMs instead of partial covariances. The partial correlation between Xi and Xj given all remaining other variables is obtained from the partial covariance matrix, very similar to the correlations matrix. The partial correlation between Xi and j given all remaining other variables is defined by

$$\rho \text{ij}|V\backslash i,j=-\frac{\Theta \text{ij}}{\sqrt{\Theta \text{ii}\Theta \text{jj}}}$$

where V\i,j denotes all variables associated with the network except *i* and *j*.

## B.

### Estimation of regression coefficients for the GGM

In nodewise regressions, we obtain estimates of the edges of the network by considering in turn each node as the dependent variable and the other nodes as predictors. We, therefore, preform *p* regressions with each p−1 predictors (the remaining variables), and obtain in total p(p−1) estimates, so two for each edge.

To obtain the set of connections for each node j=1,2,…,p, we use in linear regression, since the mean of a univariate conditional Gaussian is modeled by a linear combination of all other variables. We have data xkj for k=1,2,…,n independent observations and j=1,2,…,p variables or nodes in the network. We perform a regression for each node separately where node *i* is selected as the dependent variable and the other nodes j≠i are the predictors (see e.g., Bühlmann & van de Geer, 2011, Chapter 13; Hastie et al., 2015, Chapter 9). Then the least squares minimizes the squared residuals between node *i* and the predictions based on all nodes j≠i. The least squares estimates ${\hat{\beta }}_{\text{ij}}^{\text{LS}}$ for the p−1 remaining nodes j≠i is obtained by minimizing

(B.1) $$L\beta i(x)=\frac{1}{2n}\sum_{k=1}^{n}{\left(x\text{ki}-\sum_{j\neq i}x\text{kj}\beta \text{ij}\right)}^{2}$$

where the βi is the vector of coefficients (βi1,βi2,…,βi(i−1),βi(i+1),…,βip), so that the coefficient βii is not part of it. When n>p the estimates are unique and this is often the case for psychological data.

When the xkj are collinear or n<p then the least squares solution is no longer unique (Hastie et al., 2015). To render the solution unique a constraint can be implemented, like the least absolute shrinkage and selection operator (lasso, Tibshirani, 1996). The lasso can be written in terms of the least squares function and a penalty that sums all the coefficients βij. Additionally, there is a parameter, λ (Lagrange coefficient), associated with the penalty to set the strength of the penalty. The lasso is then

(B.2) $$L\beta i,\lambda (x)=\frac{1}{2n}\sum_{k=1}^{n}{\left(x\text{ki}-\sum_{j\neq i}x\text{kj}\beta \text{ij}\right)}^{2}+\lambda \sum_{j\neq i}|\beta \text{ij}|$$

This function can be minimized to obtain the solution $\hat{\beta }i,\lambda$ (see e.g., Hastie et al., 2015, Chapter 5). We often ignore the λ in notation for convenience. The λ parameter needs to be fixed by the researcher. This is often done by considering several (a 100, say) values of λ in a cross-validation procedure, that minimizes the squared prediction error. In cross-validation, the data is divided into *k* equally sized parts, where k−1 parts (training set) are used to estimate the coefficients, and the remaining part (test set) is used to predict the remaining values. The prediction error is obtained this way for each of the *k* parts, changing the training and test sets each time, so that an average cross-validation can be obtained for each value λ. The value λ is selected which has the smallest average cross validation error (see e.g., Hastie et al., 2015, Chapter 2, for an excellent discussion). Cross-validation has been shown to be lead to the correct value (Van der Vaart et al., 2006).

The assumptions for the lasso solution to be correct can be divided into two sets, depending on the goal. The goal could be to obtain an estimate $\hat{\beta }i$ such that the difference with the true parameter β is very small. This is called consistency. But consistency does not necessarily lead to correct identification of the non-zero coefficients in the true βi. It could be that the error $\hat{\beta }i-\beta i$ is small, but that there are some (small) non-zero values in the estimate $\hat{\beta }i$ which are zero in the true parameter βi; and it could be that for some values in the estimate $\hat{\beta }i$ the values are large while these values in the true parameter βi are small. The second goal for using the lasso, therefore, could be to identify those elements in the estimate $\hat{\beta }i$ that are zero only when the corresponding elements in the true parameter βi are zero. This is often referred to as variable selection consistency or sparsistency.

We say that the estimate $\hat{\beta }i$ is consistent if $||\hat{\beta }i-\beta i||2$ tends to 0 in probability as the number of observations n→∞, where $||a||2={\left(a_{1}^{2}+a_{2}^{2}+\cdots +a_{p}^{2}\right)}^{1/2}$ is the Euclidean norm. (Convergence in probability means that, for any ε>0, $P(||\hat{\beta }i-\beta i||2>\varepsilon )\to 0$ as n→∞.) For consistency of the lasso problem above the assumptions are that the true parameter vector is sparse and the remaining variables (all except Xi) are not highly correlated. Sparsity can be defined as the number *s* of non-zero coefficients in the true parameter vector βi. In more general settings than we discuss here, where possibly n<p, this assumption is more difficult (see Hastie et al., 2015, Chapter 11, for an excellent discussion). In the case where n>p, we basically require that the regressors (all variables except Xi) are not too highly correlated, i.e., are not collinear. So, we obtain consistency, i.e., $||\hat{\beta }i-\beta i||2\to 0$ as n→∞, if the following holds:

- *Sparsity* The true parameter βi has at most *s* (much smaller than *n*) non-zero coefficients.
- *Non-collinearity* The correlation between any variables (other than Xi) is strictly smaller than 1.

A conservative sparsity factor can be expressed in terms of the number of observations *n* and the number of variables *p*: $s=\sqrt{n/\;\text{log}\;(p)}$ (Bühlmann et al., 2014). This is a heuristic value that can be used as an indication of the sparsity.

To obtain the more subtle result that *each element* of $\hat{\beta }i$ is close the corresponding element of βi (and not just in norm $||\hat{\beta }i-\beta i||2$), i.e., variable selection consistency, two additional assumptions are required. The first is about the correlation between the regressors: the correlation between the variables that have a connection and those that do not have a connection with the dependent variable must be small. This makes sense because two regressors, where one is connected to the dependent variable and the other is not (see Figure 1(b) with X3 and X4), cannot have a correlation nearly 1 themselves. The final assumption is about the size of the ‘signal’. It assumes that the smallest non-zero value in the true parameter βi is (in absolute value) larger than some small value βmin>0.

c. *Incoherence* The correlation between unconnected variables and connected variables to Xi (the dependent variable) is at most some value γ<1.
d. *Beta-min* The smallest value in βi (in absolute value) is at least >βmin.

The beta-min assumption can be characterized $\beta \text{min}>\sqrt{\;\text{log}\;(p)/n},$ giving an indication of the lower bound on the size of the coefficients (Bühlmann et al., 2014). With these assumptions and a penalty parameter $\lambda >\sqrt{\;\text{log}\;(p)/n},$ it can be shown (Hastie et al., 2015, Theorem 11.3) that with high probability the lasso obtains

1. *Few false positives* The set of zero coefficients in the estimate $\hat{\beta }i$ is at least as large as the set of zero coefficients in the true parameter $\beta_{i}.$
2. *Many true positives* The set of non-zero coefficients in the estimate $\hat{\beta }i$ corresponds to the non-zero coefficients in the true parameter βi for those values that are >βmin.

These results have appeared in the literature in various forms. See for more details, e.g., Meinshausen and Bühlmann (2006), Wainwright (2009), Bühlmann and van de Geer (2011) and Bühlmann et al. (2013).

## C.

### p-values and the Bonferroni-Holm procedure

For each parameter estimate ${\hat{\beta }}_{\text{ij}}^{\text{LS}}$ (the least squares estimate, see Appendix B), we have that the distribution is normal and we have a standard error estimate $\text{se}(\hat{\beta }\text{ij})$ (see Appendix G). The null and alternative hypotheses are, respectively,

H0,ij:βij=0 versus Ha,ij:βij≠0

The statistic to test these hypotheses is $\hat{z}\text{ij}={\hat{\beta }}_{\text{ij}}^{\text{LS}}/\text{se}({\hat{\beta }}_{\text{ij}}^{\text{LS}}).$ Let *Z* be a random variable from the standard normal distribution. Then the two-sided *p*-value for $\hat{z}\text{ij}$ is

$$p\text{ij}=2P(|Z|\geq |\hat{z}\text{ij}|)=2(1-P(|Z|\leq |\hat{z}\text{ij}|))$$

To control the false positive rate across multiple hypothesis tests, we use the Bonferroni-Holm procedure for multiple testing correction (Hochberg & Tamhane, 1987). The Bonferroni-Holm (BH) procedure is as follows. Set the level α and denote by *m* the number p(p−1) of tests for all network edges (obtained from nodewise regressions, see Appendix B).

1. Obtain all required *p*-values pij for i,j=1,2,…,p and i≠j.
2. Order the *p*-values from small to large, so that p(11)≤p(12)≤⋯, where p(11) is the smallest *p*-value and p(12) the second smallest *p*-value, etc. Call the corresponding null hypotheses H0,(11), H0,(12) etc.
3. Test the the smallest *p*-value at the Bonferroni level α/m, i.e., p(11)<α/m.
4. If H0,(11) is rejected, then move to the next ordered *p*-value and test this at p(12)<α/(m−1). Continue testing H0,(ij) at
  $$p\text{ij}<\frac{\alpha }{m-i-j+1}$$
  for i≠j (because there are no self-loops in the network).
5. If H0,(ij) is not rejected, then stop. All remaining *p*-values will be larger than the corrected level because they are larger.

A useful way to rewrite the level at which is tested in the above BH-procedure is by setting the level α and using corrected *p*-values (this is done in many R-packages). Then, we have the corrected *p*-value for the null hypothesis H0,ij, with i≠j,

$$p_{\text{ij}}^{\text{BH}}=p\text{ij}(m-i-j+1)$$

where m=p(p−1), the number of all regression coefficients from the nodewise regressions.

## D.

### Multi sample-split

The procedure identified in Meinshausen et al. (2009) to aggregate the *K p*-values and make a single decision, uses an original correction for a type of empirical quantile function to upper bound the false positive rate. This quantile function is necessary in order to determine what cutoff to use in the distribution of *K* obtained *p*-values so that the decision to reject incorrectly remains at the correct level.

Before defining the empirical γ-quantile function introduced by Meinshausen et al. (2009), we give some definitions. The empirical distribution function gives the cumulative probability up to some γ∈(0,1), and the empirical quantile function gives the smallest observed *p*-value (in this setting) with at least probability γ. Note that the domain and range of the empirical distribution and quantile functions are the same because we are dealing with *p*-values. Let $\hat{F}j$ be the empirical distribution function for the *j*th *p*-value, based on *K* half-splits, i.e.,

$$\hat{F}j(\gamma )=\frac{1}{K}\sum_{k=1}^{K}1\{p_{j}^{k}\leq \gamma \}$$

This represents the proportion of *p*-values that are not larger than γ. The inverse mapping is the empirical quantile function

$$Qj(\gamma )=\text{min}\{p_{j}^{k}:\hat{F}j(\gamma )\geq \gamma \}$$

which gives the first *p*-value such that $\hat{F}j(\gamma )\geq \gamma .$ For example, with γ=0.5 we obtain the median, and with γ=0.25 we obtain the first quartile, usually denoted by Q1. The γ-quantile function is defined by Meinshausen et al., (2009)

$$Q_{j}^{M}(\gamma )=\text{min}\{p_{j}^{k}/\gamma :\hat{F}j(\gamma )\geq \gamma \}$$

For example, again with γ=0.5, this gives the value two times the median of the *p*-values obtained over *K* splits. Meinshausen et al. (2009, Theorem 3.2) show that the corrected *p*-value is obtained for a lower bound (usually γmin=0.05) with

$$p_{j}^{M}=(1-\;\text{log}\;\gamma \text{min})\underset{\gamma \in (\gamma \text{min},1)}{\text{min}}Q_{j}^{M}(\gamma )$$

is correct in the sense that the false positive rate is not exceeded. The correction 1− log γmin with γmin=0.05 is approximately 3.996. This corrected *p*-value, obtained from the set of *K p*-values, one for each half-split, can be considered as an adaptive control over the *K p*-values with the function (3.996/α)ϕ, where ϕ is a value in (0,1) and α is the level.

## E.

### Desparsified lasso

The desparsified lasso modifies the original lasso estimate so that a small value is added or subtracted. This small value is obtained from the residuals. By doing so, the estimated values that are exactly 0 will no longer be exactly 0. Hence, the problem is avoided of the lasso parameter estimate distribution, which is discontinuous and may have probability one on the value 0 (see Figure 2).

We follow the desparsified lasso described in van de Geer et al. (2014) and Dezeure et al. (2015). We let Xi be the dependent variables and so require a lasso estimate $\hat{\beta }i,$ which is the vector of coefficients for all j≠i. First, we perform a lasso regression on one of the regressors Xj, given the remaining variables, that is, all variables except Xi and Xj. Then with this lasso estimate $\hat{\gamma }j$ we form the residual Zj, obtained by subtracting the prediction with the lasso estimate $\hat{\gamma }j$ from the variable Xj. We then obtain the desparsified lasso for parameter βij as

(E.1) $${\hat{\beta }}_{\text{ij}}^{d}=\hat{\beta }\text{ij}+\frac{\sum_{m=1}^{n}e\text{mi}Z\text{mj}}{\sum_{m=1}^{n}X\text{mj}Z\text{mj}}+Un$$

where $\hat{\beta }\text{ij}$ is the lasso estimate, emi is the residual of the *m*th observation of variable *i* (dependent variable), and Un denotes a random variable that tends to 0 in probability at the rate of $\frac{1}{\sqrt{n}}.$ van de Geer et al. (2014) have shown that if Un is negligible then (E.1) is approximately normally distributed, given certain assumptions. This is because the middle term in (E.1) is a function of the residuals emi which are assumed to be normal (see Section 3). Hence, the standard error can be obtained to determine the *p*-values and confidence intervals. The assumptions required for the desparsified lasso are the first two assumptions (*a*) and (*b*) in Appendix B.

## F.

### Bootstrap of the desparsified lasso

Here, we clarify the steps in the algorithm for the bootstrap in Section 4.3. In step (1), we basically resample the set of labels {1,2,…,n} for the observations and then select those observations xki for all i=1,2,…,p and call the resampled version $x_{\text{ki}}^{⋆}$ for all i=1,2,…,p. In step (2), we obtain a network by applying the desparsified lasso to the resampled observations $x_{\text{ki}}^{⋆}.$ The distribution of the estimates ${\hat{\beta }}_{\text{ij}}^{⋆}$ are then guaranteed by the results of the desparsified lasso. Then, finally, after having repeated (1) and (2) *K* times, in step (4) we find for each parameter βij the empirical distribution based on the *K* bootstrap samples $x_{\text{ki}}^{⋆}$ for all i=1,2,…,p. The empirical distribution function for the *K* bootstrap samples ${\hat{\beta }}_{\text{ij}}^{⋆}$ is

$${\hat{F}}_{\text{ij}}^{⋆}(\beta )=\frac{1}{K}\sum_{k=1}^{K}1\{{\hat{\beta }}_{\text{ij}}^{⋆,k}\leq \beta \}$$

for some value β. The *p*-value is then calculated by determining with the original sample with estimate $\hat{\beta }\text{ij}$ the value ${\hat{F}}_{\text{ij}}^{⋆}(\hat{\beta }\text{ij}).$ It can be seen that the error for the bootstrap depends on the number of bootstraps *K* (see Davison & Hinkley, 1997, Chapter 2, for an excellent discussion on this). In the order of 500 bootstrap, resamples is generallly considered adequate. The assumptions for the bootstrap are the same as for the desparsified lasso, so, sparsity (*a*) and no collinearity (*b*).

## G.

### Confidence intervals

Two cases will be discussed here, without the lasso penalty (least squares) and with the lasso penalty (see Appendix B), where we use the desparsified lasso described in Appendix E. We first discuss the case without the lasso penalty and where we have n>p, so that we have more observations than parameters.

To obtain a confidence interval (and *p*-values), we require that the distribution of the estimate ${\hat{\beta }}_{\text{ij}}^{\text{LS}}$ is normal, from which we obtain the mean (the true value βij) and the standard error (the standard deviation of this sampling distribution, see e.g., Bilodeau & Brenner, 1999). The distribution of ${\hat{\beta }}_{\text{ij}}^{\text{LS}}$ is normal because the residuals of the linear model are normally distributed. Let Ri be the matrix with all regressors j≠i predicting variable *i*. Consider the least squares estimate for all j≠i coefficients simultaneously

$${\hat{\beta }}_{i}^{\text{LS}}={\left(R_{i}^{⊤}Ri\right)}^{-1}R_{i}^{⊤}Xi$$

We know that the distribution is normal because we know that Xi is normally distributed. For the linear model, we obtain from this the mean (conditional on the remaining variables j≠i)

$$E({\hat{\beta }}_{i}^{\text{LS}})={\left(R_{i}^{⊤}Ri\right)}^{-1}R_{i}^{⊤}E(Xi)=\beta i$$

So that we recover in expectation the true parameters ${\hat{\beta }}_{\text{ij}}^{\text{LS}}$ for j≠i. We also obtain the covariance matrix

$$\text{var}({\hat{\beta }}_{i}^{\text{LS}})={\left(R_{i}^{⊤}Ri\right)}^{-1}R_{i}^{⊤}\text{var}(Xi)Ri{\left(R_{i}^{⊤}Ri\right)}^{-1}=\sigma_{e}^{2}{\left(R_{i}^{⊤}Ri\right)}^{-1}$$

where we assumed that the variable Xi has residual variance $\sigma_{e}^{2}$ (see Section 3). The standard error, denoted by $\text{se}({\hat{\beta }}_{\text{ij}}^{\text{LS}}),$ can be obtained from the covariance matrix of the parameter estimates. Taking the square root of the diagonal elements of this matrix gives the standard error. Having obtained the standard errors $\text{se}({\hat{\beta }}_{\text{ij}}^{\text{LS}}),$ we obtain a 95% confidence interval for parameter βij using the normal approximation

(G.1) $${\hat{\beta }}_{\text{ij}}^{\text{LS}}-1.96\text{se}({\hat{\beta }}_{\text{ij}}^{\text{LS}})<\beta \text{ij}<{\hat{\beta }}_{\text{ij}}^{\text{LS}}+1.96\text{se}({\hat{\beta }}_{\text{ij}}^{\text{LS}})$$

such that

(G.2) $$P{\hat{\beta }}_{\text{ij}}^{\text{LS}}\left[{\hat{\beta }}_{\text{ij}}^{\text{LS}}-1.96\text{se}({\hat{\beta }}_{\text{ij}}^{\text{LS}})<\beta \text{ij}<{\hat{\beta }}_{\text{ij}}^{\text{LS}}+1.96\text{ se}({\hat{\beta }}_{\text{ij}}^{\text{LS}})\right]=0.95$$

Because the estimates ${\hat{\beta }}_{\text{ij}}^{\text{LS}}$ are a linear function of the data, we obtain that for multivariate normal random variables that the estimates have a normal distribution. Furthermore, the standard error $\text{se}({\hat{\beta }}_{\text{ij}}^{\text{LS}}),$ the standard deviation of the sampling distribution of ${\hat{\beta }}_{\text{ij}}^{\text{LS}},$ can be calculated.

The second case, where we use the lasso penalty to obtain the estimate ${\hat{\beta }}_{\text{ij}}^{d},$ we use the desparsified lasso. The desparsified lasso is used because we require that the sampling distribution of the estimates ${\hat{\beta }}_{\text{ij}}^{d}$ is approximately normal. We know that the desparsified lasso yields estimates with an approximately normal distribution, as discussed in Appendix E. Additionally, the desparsified lasso obtains the standard error, so that we can calculate the confidence interval in the same way as shown above.

## H.

### Simulation details

To generate the partial covariances Θij, we first obtained an adjacency matrix with the function erdos.renyi.game (in the package igraph). The adjacencies Aij are 0 indicating no edge (i,j) in the network and 1 if there is an edge (i,j) in the network. Subsequently, we generated p(p−1)/2 random partial covariance coefficients from a uniform distribution with values between 0.1 and 0.8, and collected these in Uij. Then we defined the partial covariances as Θij=AijBij. The diagonal elements Θii were defined as the sum of the row coefficients: $\Theta \text{ii}=\sum_{j=1}^{p}\Theta \text{ij}.$ This last step ensured that Θ was nonsingular.

In the random network generation, we set pe to 0.2, yielding between 2 and 9 connections for each node, and we set pe to 0.4 yielding between 5 and 12 connections for each node. Using the sparsity $s=\sqrt{n/\;\text{log}\;(p)}$ (see Appendix B) we get for n=50a maximum of 4 connections for each node, and for n=500 we obtain a maximum of 12 connections for each node. The number connections for each node in the generated network could violate sparsity *s*. In Table 2, we provide the empirical distribution function for the number of nodes that violate the sparsity assumption.

Using the heuristic of the beta-min value $\beta \text{min}>\sqrt{\;\text{log}\;(p)/n}$ (Bühlmann et al., 2014), we can also determine the percentage of edges out of the p(p−1)/2 edges (190 for our case of p=20 nodes) do not satisfy the beta-0min assumption. Table 2 shows the percentages of edges that violate the beta-min assumption (*d*) from Appendix B. For n=50 the percentage is about 30, and for n=500 the percentage is 0.

The multivariate normal data were generated using the eigenvalue decomposition of $\Theta^{-1}=\Sigma =V\Lambda V^{⊤},$ where Λ is a diagonal matrix with eigenvalues Λ1,Λ2,…,Λp, all positive, and eigenvectors V1,V2,…,Vp as columns of *V*. A square root of Σ is then defined as $\sqrt{\Sigma }=V\sqrt{\Lambda }V^{⊤},$ with $\sqrt{\Lambda }$ the diagonal matrix with elements $\sqrt{\Lambda i}.$ Standard normally distributed data were generated independently and collected in the n×p matrix *Z*. To obtain the GGM with the specified structure Σ, we constructed data $X=Z{\sqrt{\Sigma }}^{⊤}.$

The following settings were used for retrieving the *p*-values with each of the methods. For the multi sample-split, we used the recommended B=50 for the number of splits and a split fraction of 0.5 (Dezeure et al., 2015; Meinshausen et al., 2009). For the desparsified lasso, we selected the penalty parameter λ using 10-fold cross-validation; we used 10-fold cross-validation for the lasso without *p*-values as well. And, finally, for the bootstrap (using the desparsified lasso), we used the same settings as for the desparsified lasso and used B=1000 resamples for the bootstrap. For each of the three methods, we used the Bonferroni-Holm correction to obtain corrected *p*-values. To determine the presence or absence of an edge, we used the *and*-rule.

## I.

### Additional simulation results: Specificity and conditional coverage

Figure I.1. displays additional results of the simulation study reported in the main text. The first row shows the specificity, the second row the coverage for absent edges, and the third row the coverage for present edges.

**Table 1.** The table provides an overview of the assumptions that are required for each of the three methods of obtaining *p*-values, the lasso, the graphical lasso (glasso) and OLS.

	Method										
Assumptions	OLS	lasso	Multisplit	Desp lasso	Boot desp	Glasso					
(*a*) sparsity	no	yes	yes	yes	yes	yes
(*b*) no collinearity	yes	yes	yes	yes	yes	yes
(*c*) incoherence	no	yes	yes	no	no	yes
(*d*) beta-min	no	yes	yes	no	no	yes

**Table 2.** Sparsity (for nodewise estimation) and beta-min violations for each of the settings in the simulation with probability of an edge pe=0.2 or 0.4. Sparsity is in the second column for each number of observations (from 50 to 500) and p=20 nodes. In the third and fourth columns, the number of nodes in the network that violate the sparsity assumption. In the fifth column, is the heuristic beta-min value for each of the number of observations. Sixth and seventh columns are the percentage of edges that violate the beta-min assumption (i.e., are smaller than the beta-min value).

	#> Sparsity pe			%< Beta-min pe		
*n*	Sparsity	0.2	0.4	Beta-min	0.2	0.4
50	4	9	20	0.25	22	29
100	5	4	18	0.17	17	17
200	8	1	9	0.12	2	6
500	12	0	0	0.08	0	0
800	16	0	0	0.06	0	0
1000	18	0	0	0.05	0	0
